# Availability of Information about Airborne Hazardous Releases from Animal Feeding Operations

**DOI:** 10.1371/journal.pone.0085342

**Published:** 2013-12-31

**Authors:** Tyler J. S. Smith, Leonard S. Rubenstein, Keeve E. Nachman

**Affiliations:** 1 Johns Hopkins Center for a Livable Future, Johns Hopkins University, Baltimore, Maryland, United States of America; 2 Department of Environmental Health Sciences, Johns Hopkins Bloomberg School of Public Health, Baltimore, Maryland, United States of America; 3 Department of Epidemiology, Johns Hopkins Bloomberg School of Public Health, Baltimore, Maryland, United States of America; 4 Department of Health Policy and Management, Johns Hopkins Bloomberg School of Public Health, Baltimore, Maryland, United States of America; The Ohio State University, United States of America

## Abstract

**Introduction:**

Air from animal feeding operations (AFOs) has been shown to transport numerous contaminants of public health concern. While federal statutes like the Emergency Planning and Community Right-to-Know Act (EPCRA) generally require that facilities report hazardous releases, AFOs have been exempted from most of these requirements by the U.S. Environmental Protection Agency (EPA). We assessed the availability of information about AFO airborne hazardous releases following these exemptions.

**Methods:**

We submitted public records requests to 7 states overlapping with or adjacent to the Chesapeake Bay watershed for reports of hazardous releases made by AFOs under EPCRA. From the records received, we calculated the proportion of AFOs in each state for which ≥1 reports were available. We also determined the availability of specific types of information required under EPCRA. The numbers of AFOs permitted under the Clean Water Act (CWA) or analogous state laws, as determined from permitting databases obtained from states, were used as denominators.

**Results:**

We received both EPCRA reports and permitting databases from 4 of 7 states. Across these 4 states, the mean proportion of AFOs for which ≥1 EPCRA reports were available was 15% (range: 2-33%). The mean proportions of AFOs for which the name or identity of the substance released, ≥1 estimates of quantity released, and information about nearby population density and sensitive populations were available were 15% (range: 2-33%), 8% (range: 0-22%), and 14% (range: 2-8%), respectively.

**Discussion:**

These results suggest that information about the airborne hazardous releases of a large majority of AFOs is not available under federal law in the states that we investigated. While the results cannot be attributed to specific factors by this method, attention to multiple factors, including revision of the EPA’s exemptions, may increase the availability of information relevant to the health of populations living or working near AFOs.

## Introduction

Industrial food animal production is the dominant model of meat, poultry, dairy and egg production in the United States [1,2]. The model typically involves housing thousands to hundreds of thousands of animals in confinement at a single site, and results in the accumulation of large quantities of animal waste. Depending on the type of animal produced and the waste management system utilized, waste may be stored on-site for extended periods of time or piped into large cesspits known as “lagoons” [3]. The U.S. Environmental Protection Agency (EPA) terms these facilities “animal feeding operations” (AFOs) [4]. As defined by the Agency, AFOs are sites where non-aquatic animals are confined for ≥45 days/year and where crops or vegetation are not sustained or stored in the normal growing season [4]. The largest AFOs are known as “concentrated animal feeding operations” (CAFOs) and are classified as such based on the number of animals on-site (the number varies based on the type of animal) [4].

Research has shown that this model of food animal production and associated methods of waste management can contribute to compromised air quality [5,6]. Air within and emanating from AFOs has been shown to transport numerous contaminants of public health concern, including gases, particulate matter, bacterial and viral pathogens, endotoxins, and animal dander [7-13]. Epidemiologic investigations have illustrated relationships between residential proximity to AFOs and respiratory effects [14-16], mental health outcomes [17-19], and other quality-of-life-related impacts [20,21]. 

Federal statutes generally require that facilities report estimates of their emissions if these emissions exceed certain rates. Section 103 of the Comprehensive Environmental Response, Compensation, and Liability Act (CERCLA) requires facilities that release one or more “hazardous substances” in excess of “reportable quantities” (RQs, expressed as rates in pounds per day [lbs/day]) to immediately notify the National Response Center, unless the release is permitted under another federal statute [22]. Section 304 of the Emergency Planning and Community Right-to-Know Act (EPCRA) similarly requires facilities that release one or more “extremely hazardous substances” in excess of RQs to immediately notify state and local emergency management committees [23]. EPCRA requires these committees to make reports of hazardous releases available to the public [23]. 

The substances released by AFOs that are designated as hazardous under CERCLA [24] and/or extremely hazardous under EPCRA [25] include ammonia, hydrogen sulfide, and multiple volatile organic compounds (VOCs) [26]. From 2001-2006, litigation by environmental advocates and, in two cases, the EPA confirmed that the reporting requirements of CERCLA Section 103 and EPCRA Section 304 applied to AFOs, resulting in penalties for multiple operations that released ammonia in excess of its RQ and did not report their releases [27]. (Agricultural operations, including AFOs, are not required to report to the Toxic Release Inventory established by EPCRA Section 313 [28].) Industry trade associations and some members of Congress subsequently proposed legislation to exempt AFOs from these requirements and petitioned the EPA to exempt AFOs by regulation [27,29]. The legislation was not enacted; despite this, the agency subsequently exempted most AFOs from the requirements of both statutes.

In 2005, the EPA and industry trade associations initiated the National Air Emissions Monitoring Study (NAEMS) to develop emissions-estimating methodologies (EEMs) for AFO air emissions [30,31]. More than 13,000 AFOs agreed to fund NAEMS under a 2005 consent agreement with the agency. In exchange, the EPA agreed not to sue participating AFOs for civil violations of CERCLA and EPCRA reporting requirements that occur before the EPA publishes final EEMs [32,33].. In 2008, the EPA finalized a rule that indefinitely exempted all airborne hazardous releases from animal waste at farms (including AFOs) from CERCLA reporting requirements [34-36]. The same rule also indefinitely exempted such releases from all but Large CAFOs (the largest CAFOs, which in turn are the largest AFOs) from EPCRA reporting requirements [34,35]. In its preamble to the rule, the EPA also stated that reportable releases by Large CAFOs generally qualify for reduced EPCRA reporting requirements that were developed for reporting continuous releases [34,36]. Under these requirements, Large CAFOs must report releases to emergency management committees once by telephone and once in writing within 30 days of the notification by telephone; subsequent releases that are similar to the initial reported release are not reportable [37]. 

The 2005 agreement and the 2008 rule were both in effect as of November 2013. The combined effect of the agreement and the rule is that, of all AFOs, only Large CAFOs currently are required to report under EPCRA and the EPA will not enforce this requirement against Large CAFOs that are participating in NAEMS. Large CAFOs that do report under EPCRA use an abbreviated reporting format designed for continuous releases. It is generally assumed that these changes have limited the availability of information about AFO hazardous releases [31,36], but this has not been confirmed. 

**Figure 1:**
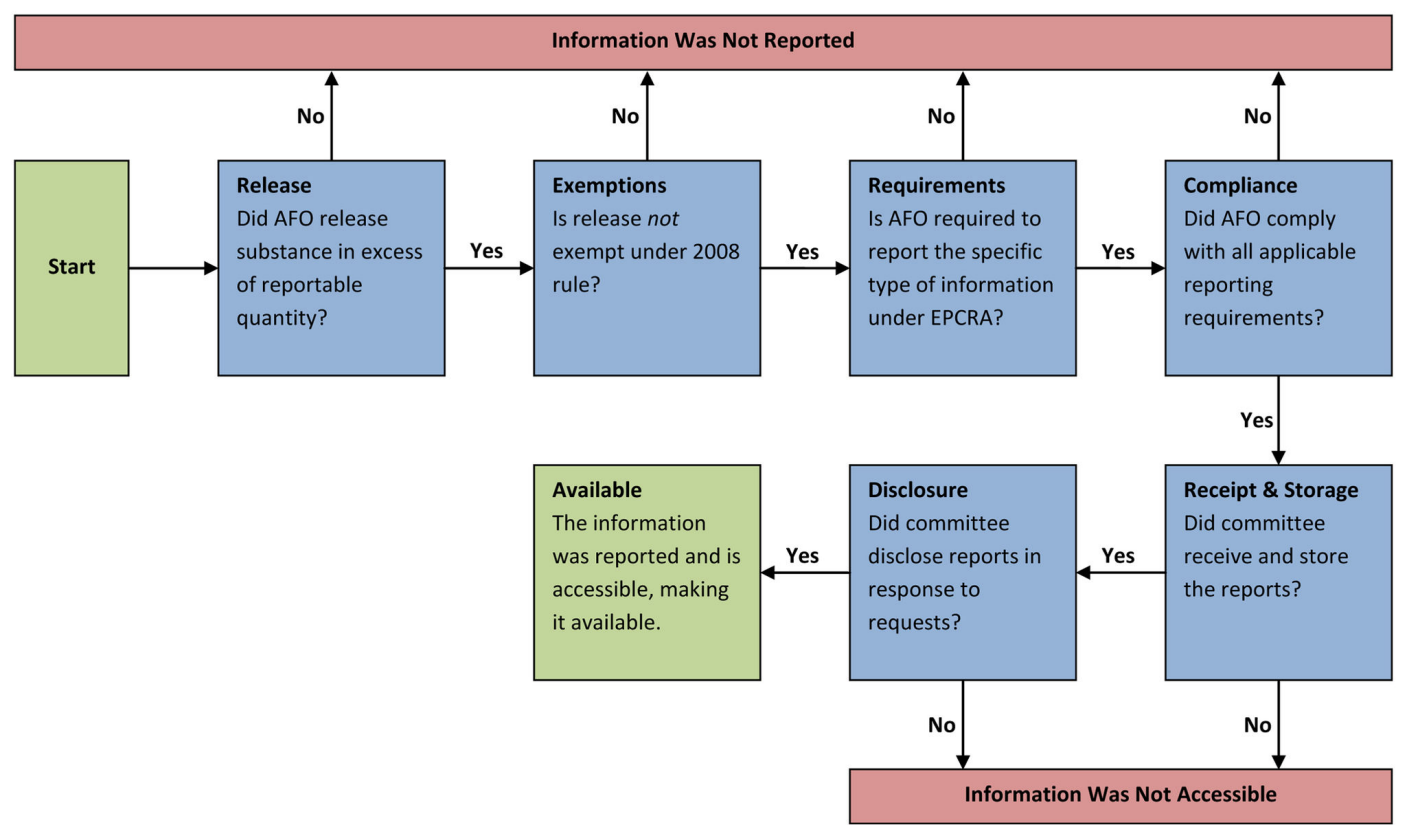
Is a specific type of information about AFO airborne hazardous releases available under EPCRA? The availability of information encompasses two elements: whether information was reported and whether reported information is accessible to the public. These elements in turn depend on multiple factors. Reporting factors are determined by the U.S. Environmental Protection Agency via rulemaking. Access factors are determined by the state and local emergency management committees that receive reports under EPCRA.

The concept of availability encompasses both whether *information was reported* by an AFO and whether the reported *information is accessible* to the public. Both elements of availability in turn depend on multiple factors (Figure 1). CERCLA and EPCRA reporting reflect whether an AFO releases a hazardous or extremely hazardous substance in excess of its RQ, whether the release is exempt from reporting under the 2008 rule, and whether the AFO complies with applicable reporting requirements. Notably, amnesty from civil prosecution under the 2005 agreement may influence AFO compliance. If an AFO complies fully, the types of information reported will vary with regulatory requirements; for example, reporting continuous releases under EPCRA requires AFOs to provide less information than does reporting other releases. Availability also requires access to reported information, which depends on proper receipt and storage of reports by emergency management committees, as well as disclosure of these reports in response to public records requests.

The purpose of this study was to assess availability of information concerning airborne hazardous releases from AFOs under EPCRA following the 2005 agreement and promulgation of the 2008 rule. We determined the proportions of AFOs for which ≥1 EPCRA reports were available and the proportions of AFOs for which specific types of information required under EPCRA regulations were available. We discuss the feasibility of assessing human exposure to hazardous releases using the information available under EPCRA and options for assuring the availability of information needed to assess exposure.

## Methods

We investigated the availability of information under EPCRA Section 304 because this section contains the only requirement under federal law (and therefore applicable across multiple states) to report airborne hazardous releases that currently applies to AFOs.

**Figure 2:**
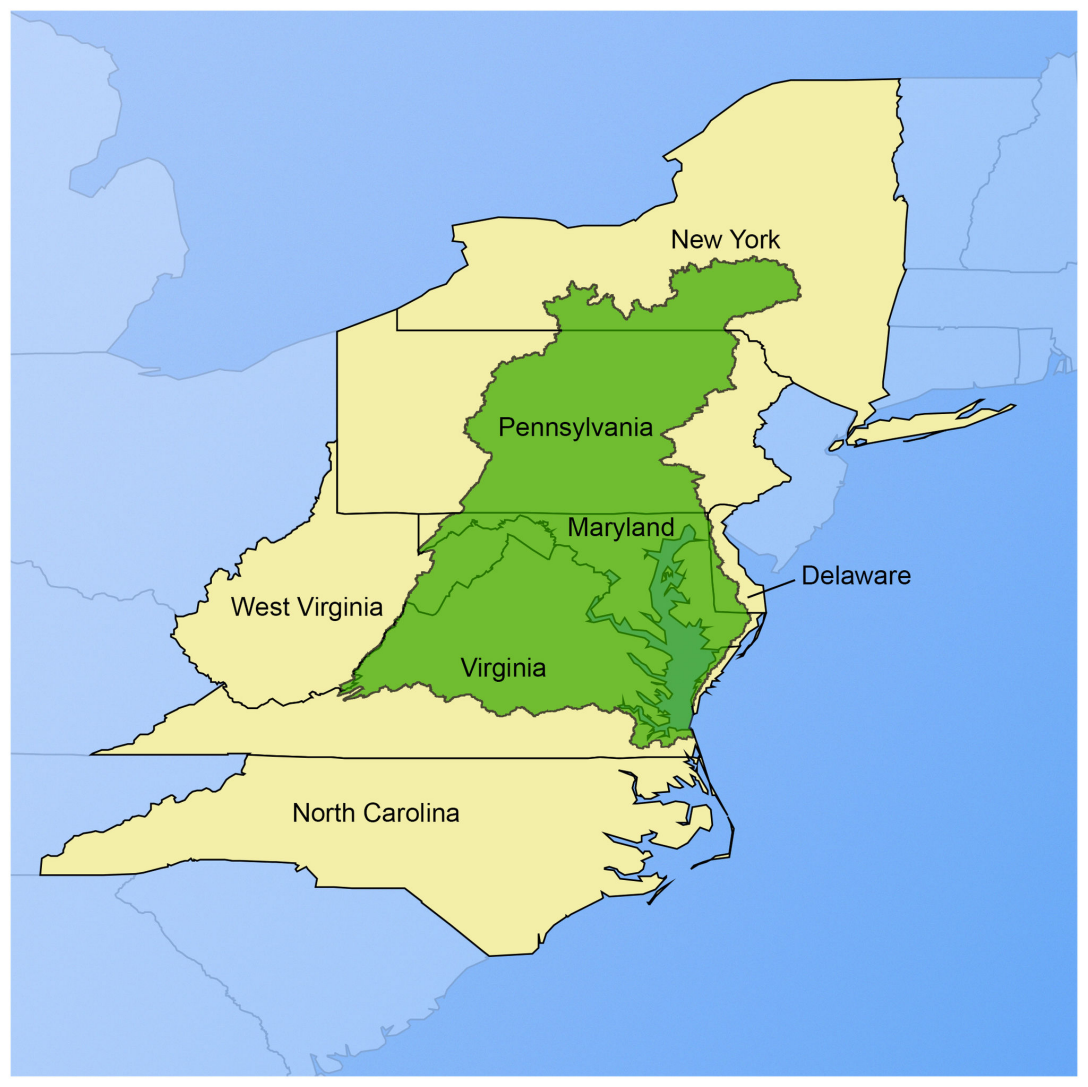
Map of included states and the Chesapeake Bay watershed. We included states (shaded yellow): 6 states that overlap with the Chesapeake Bay watershed (shaded green) and North Carolina.

Between September 2010 and February 2012, we submitted public records requests to the six states that overlap with the Chesapeake Bay watershed (Delaware [DE], Maryland [MD], New York [NY], Pennsylvania [PA], Virginia [VA], and West Virginia [WV]) and North Carolina (NC) (Figure 2). The requests sought reports submitted by AFOs under the 2008 rule, as described by an EPA document [38]. The Chesapeake Bay watershed was chosen because the high density of AFOs in this region has impacted environmental quality [39]. NC is the second-largest swine producer in the U.S. [40], and was included so that production of most major food animal types (i.e., poultry, swine, and dairy cattle) was represented; NC was chosen as the swine-producing state because it is contiguous with the other states investigated.

Multiple forms are available to assist AFOs in reporting continuous releases under EPCRA, including forms developed by the EPA, state governments, and industry trade associations [41-43]. AFOs may choose which forms to use for their submissions. We developed a database of EPCRA forms received in response to our requests. For each form, we recorded the name of the AFO and the name of the AFO owner or operator. Because some AFOs submitted multiple forms, we matched the forms by the names of the AFO and/or AFO owner or operator and consolidated records to create one record per AFO. In some cases, releases from multiple AFOs were reported jointly on a single form. From these forms, we created separate records for each AFO. The end-result was a database with one record per AFO for which ≥1 forms was received. We recorded which forms were submitted by each AFO.

We then determined the proportion of AFOs in each state for which ≥1 EPCRA reports were submitted to state or local emergency management committees. For the numerators, we used the number of AFOs in each state for which we received ≥1 forms. To develop denominators, we obtained databases of AFOs permitted under the Clean Water Act (CWA) or similar state laws (such as the Virginia Pollution Abatement Permit Regulation and Pollutant Discharge Elimination Programs [44]) from states with active permitting programs. We divided the numerator for each state by the number of permitted AFOs in that state to obtain the proportion. Because permitting databases only include permitted AFOs, denominators derived from these databases likely are underestimates of the number of AFOs in each state; consequentially, the proportions calculated with these denominators likely are overestimates of the true percentage of AFOs that have reported airborne hazardous releases.

We then assessed availability of 6 types of information, including the identity of the substance released, estimates of the quantity released, and characteristics of potentially exposed populations, as required by EPCRA regulations. The identity of the substance released must be reported by its chemical name and Chemical Abstracts Service (CAS) number [37,45]. For continuous releases, estimates of the quantity released must be reported in two ways: estimates of the upper and lower bounds on the quantity released over any 24-hour period during the previous year and an estimate of the total amount released during the same year [37,45]. The quantities of some AFO releases have been estimated by multiplying the number of animals of a particular species at an AFO by a constant “emissions factor” for a given substance, animal species, and time period (e.g., *x* lbs of ammonia per broiler chicken per 24 hours) [26]. Multiple forms available to AFOs were designed to record the species and number of animals at an AFO in addition to or in lieu of actual estimates of the quantity released, and we assessed availability of this information as well. The only required information regarding potentially exposed populations is the population density within a one-mile radius of the AFO and the identity and the location of buildings that are indicative of sensitive populations (e.g., elementary schools and nursing homes) [37,45]. 

To obtain the numerator for each type of information, we counted the number of AFOs in each state for which we received ≥1 EPCRA forms that were designed to record that information. We divided each numerator by the number of permitted AFOs in the state to obtain the proportion.

## Results

We received EPCRA reports from 5 of 7 states (see Table 1). NY reported that the requested records could not be found and NC did not respond substantively to the request despite our follow up by email and phone. The permitting databases used to estimate the number of AFOs in each state were obtained from every state except WV, which was still developing its permitting program for AFOs and had not permitted any AFOs as of June 2013. For the 4 states from which EPCRA reports were received and that provided permitting databases (DE, MD, PA, and VA), we calculated the proportions of AFOs for which ≥1 EPCRA reports were available. The mean proportion across the 4 states was 15% (range: 2-33%). 

**Table 1:** Number and proportions of AFOs for which ≥1 EPCRA reports were available, by state.

**State**	**Permitted AFOs**	**Forms Received**	**AFOs Available - n (%)**
DE	395	64	56 (14)
MD	353	207	118 (33)
NC	2790	0	0 (0)
NY	492	0	0 (0)
PA	491	8	8 (2)
VA	912	158	100 (11)
WV	0	19	13 (-)
Mean^1^			**15%**

Permitted AFOs is the number of AFOs permitted under the Clean Water Act or an analogous state law. Forms Received is the number of EPCRA forms received in response to our requests. Some AFOs submitted multiple forms: n is the number of AFOs for which ≥1 EPCRA forms were received. % is n divided by Permitted AFOs. ^1^Mean only includes states for which we received both EPCRA reports and permitting databases (DE, MD, PA, and VA, but not NC, NY, or WV).

We then determined the availability of the aforementioned types of information in the 4 states (see Table 2). The mean proportion of AFOs for which the name or CAS number of the substance released was available was 15% (range: 2-33%). The mean proportion of AFOs for which the presence of sensitive populations within a 1-mile radius was available was 14% (range: 2-32%) and the mean proportion for which population density was available was likewise 14% (range: 2-32%).

**Table 2:** Number and proportions of AFOs for which specific types of information were available, by state.

	**DE**	**MD**	**NC^1^**	**NY^1^**	**PA**	**VA**	**WV^1^**	**Mean^1^**
	**n (%)**	**n (%)**	**n (%)**	**n (%)**	**n (%)**	**n (%)**	**n (%)**	**%**
**Name and/or CAS number of substance(s) released**	56 (14)	118 (33)	0 (0)	0 (0)	8 (2)	100 (11)	13 (-)	15
**Population density within 1-mile radius**	56 (14)	112 (32)	0 (0)	0 (0)	8 (2)	79 (9)	13 (-)	14
**Sensitive populations within 1-mile radius**	56 (14)	112 (32)	0 (0)	0 (0)	8 (2)	79 (9)	13 (-)	14
**Bounds on quantity released in any 24-hour period in past year**	28 (7)	28 (8)	0 (0)	0 (0)	8 (2)	4 (0)	1 (-)	4
**Quantity released in past year**	28 (7)	28 (8)	0 (0)	0 (0)	8 (2)	0 (0)	1 (-)	4
**Number and species of animals at AFO**	28 (7)	62 (18)	0 (0)	0 (0)	0 (0)	0 (0)	0 (-)	6
**≥1 estimate of quantity released**	28 (7)	76 (22)	0 (0)	0 (0)	8 (2)	4 (0)	1 (-)	8

n is the number of AFOs in that state for which ≤1 EPCRA forms designed to record the corresponding type of information were available. % is n divided by the number of Permitted AFOs in that state (see Table 1). ^1^Mean only includes states for which we received both EPCRA reports and permitting databases (DE, MD, PA, and VA, but not NC, NY, or WV).

Estimates of the upper and lower bounds on the quantity released in any 24-hour period were available, on average, for 4% of AFOs across the 4 states (range: 0-8%). The mean availability of estimates of the total amount released annually was likewise 4% (range: 0-8%). Because the species and number of animals at an AFO also has been used to estimate the quantity released, we calculated the proportion of AFOs for which both of these types of information were available. The mean was 4% (range: 0-18%). The mean proportion of AFOs for which at least one of these estimates of quantity released (upper and lower bounds, amount released annually, or species and number of animals) was available was 8% (range: 0-22%).

## Discussion

We assessed the availability of information concerning AFO airborne hazardous releases under EPCRA. We were unsuccessful in obtaining EPCRA reports submitted in 2 states, indicating that this information may not be available in these states. Across 4 other states, the mean proportion of AFOs for which we were unable to obtain EPCRA reports was 85% (range: 67-98%). The availability of quantitative information was even more limited: at least one estimate of the quantity released was not available for an average of 92% (range: 78-100%) of permitted AFOs across 4 states. The findings suggest that information about the hazardous releases of a large majority of AFOs is not available in the states that we investigated. The included states contain large numbers of poultry, swine, and dairy cattle operations. A limitation of our study was that it did not include a major beef-cattle state. 

The requirement to obtain a CWA or analogous permit and AFO compliance with permitting requirements vary by state, and it has been estimated that a majority of AFOs are not permitted [46]. Unpermitted AFOs could not be included in our denominators, as these were based on state permitting databases. The denominators therefore are underestimates of the actual numbers of AFOs by state and, accordingly, our results are likely to be overestimates of the proportions of AFOs for which information was available. There is no comprehensive database of AFOs across multiple states, limiting the development of more accurate denominators. The U.S. Government Accountability Office has recommended that the EPA develop such a database [46]. In July 2012, however, the agency withdrew a proposed rule that would have required many AFOs to report their location and characteristics regardless of whether they are required to obtain a CWA permit [47]. 

### Information via EPCRA continuous release reporting

As noted above, the concept of availability under EPCRA encompasses both whether information was reported to state and local emergency management committees and whether the reported information was accessible. We believe that the lack of availability observed was influenced more by a lack of reporting than by a lack of access, but a limitation of our study was that the method employed did not allow us to attribute the lack of availability to specific factors. Attention to the factors that influence reporting and access (Figure 1), however, may allow the EPA and emergency management committees to increase availability of information relevant to the health of populations that live or work near AFOs.

AFOs that do not release an extremely hazardous substance in excess of the corresponding RQ are not required to report under EPCRA. Information about AFO releases below RQs is not currently available under federal law. Hazardous releases below RQs may nevertheless pose a health risk to exposed populations, either singularly or through cumulative exposure to releases from multiple sources. For example, a single AFO that releases ammonia at a rate of 100 lbs/day or more would be required to report the release under CERCLA and EPCRA unless exempted. In contrast, two adjacent AFOs that release 50 lbs of ammonia per day are not required to report under either statute, although the resulting human exposure may be similar. Additionally, exposure that results from a single release of less than 100 lbs of ammonia per day may pose a health risk, depending on factors that influence human exposure and susceptibility. The RQ for a given substance is based on the intrinsic properties of that substance and not on projections of human exposure or resulting health risks [48], limiting the efficacy of the reporting requirements of EPCRA Section 304 for informing exposed communities about the risks posed by hazardous releases.

It is also likely that many otherwise reportable releases were not reported due to the 2005 agreement and/or the 2008 rule. Under the rule, only Large CAFOs are required to report under EPCRA. Under the agreement, the EPA has agreed not to sue AFOs, including Large CAFOs, that participated in NAEMS for civil violations of this and other requirements. The 2005 agreement will expire following the finalization of EEMs by the EPA [30]. The 2008 rule is indefinite, although the EPA has suggested that it may be revised [49]. A withdrawal or revision of the 2008 rule could substantially increase the number of AFOs that report airborne hazardous releases. Any revision should be accompanied by enhanced enforcement of reporting requirements, as AFO compliance with environmental statutes may be limited [46]. 

Additionally, although the accuracy of available information was not assessed in this study, it is notable that EPCRA Section 304 directs facilities to report estimates of the quantity released rather than measure releases directly. A study found that trends in estimates reported to the TRI under EPCRA Section 313 were not matched by trends in measurements taken by EPA pollution monitors, suggesting that reported estimates were inaccurate [50]. This may mean that at least some of the limited information available under EPCRA Section 304 is not accurate, which would limit its value.

Finally, even when AFOs do report releases, public records requests may not assure timely access to EPCRA reports. For example, while most states responded to our requests in a timely manner, NC never responded substantively. To assure public access to reported information, emergency management committees should consider alternative methods for releasing EPCRA reports, such as publishing reports online upon their submission.

### Alternatives to EPCRA

Despite the accumulating body of evidence linking residential proximity to AFOs and adverse health outcomes [14-21], little is known about the magnitude, temporality, and variability of community exposures to AFO hazardous releases. Even less is known about how the geographic clustering of these operations may impact air quality in agricultural communities. The development of regulations and other interventions to minimize harmful exposures would be enhanced through a clearer understanding of these processes and relationships.

The first step toward characterization of the contribution of AFOs to rural community air quality would be the establishment of pollution monitors both at AFOs and in communities. These data, paired with AFO-specific production characteristics and information about atmospheric conditions, would allow for the development of process-based models that facilitate geospatial estimation of airborne concentrations of pollutants of AFO origin. Development of the these process-based models, which incorporate data on chemical, biological and physical processes at play in AFOs, have been recommended by the EPA’s Science Advisory Board [51]. Such information would serve multiple purposes, allowing both for epidemiologic investigations aimed at characterizing the community health risks posed by AFOs and for examinations of the public health benefits offered by interventions designed to mitigate risks. 

## Conclusions

Despite literature associating AFOs with compromised air quality and residential proximity to AFOs with adverse health outcomes, availability of information concerning AFO airborne hazardous releases ranged from limited to nonexistent across the states that we examined. These data would be complemented by a national spatially-referenced database of AFO locations, which would provide supporting information regarding the clustering of these operations in rural communities. These data gaps compromise the ability of public health officials and scientists to characterize exposures and risks, and limit their ability to implement and evaluate interventions when appropriate. The lack of data also means that information on AFO hazardous releases is not available to residents of affected communities. Lowering RQs, withdrawing exemptions for AFOs, and improving public access to EPCRA reports could increase availability within the existing statutory framework. Ultimately, however, air monitoring at AFOs and in surrounding communities will be needed to more robustly characterize the public health impacts of AFO air pollution. 
